# The Impact of 52-Week Single Inhaler Device Triple Therapy versus Dual Therapy on the Mortality of COPD Patients: A Systematic Review and Meta-Analysis of Randomized Controlled Trials

**DOI:** 10.3390/life12020173

**Published:** 2022-01-25

**Authors:** Chih-Cheng Lai, Chao-Hsien Chen, Kuang-Hung Chen, Cheng-Yi Wang, Tsan-Ming Huang, Ya-Hui Wang, Hao-Chien Wang

**Affiliations:** 1 Department of Internal Medicine, Kaohsiung Veterans General Hospital, Tainan Branch, Tainan 710, Taiwan; n261@mail.vhyk.gov.tw; 2 Division of Pulmonary, Department of Internal Medicine, MacKay Memorial Hospital, Taipei 104, Taiwan; stardust.6262@mmh.org.tw; 3 Department of Medicine, MacKay Medical College, New Taipei City 252, Taiwan; 4 Department of Internal Medicine, National Taiwan University Hospital and College of Medicine, National Taiwan University, Taipei 100, Taiwan; jetcgh@mospital.com (K.-H.C.); haochienwang@ntu.edu.tw (H.-C.W.); 5 Department of Internal Medicine, Cardinal Tien Hospital and School of Medicine, College of Medicine, Fu Jen Catholic University, New Taipei City 231, Taiwan; 6 Medical Research Center, Cardinal Tien Hospital and School of Medicine, College of Medicine, Fu Jen Catholic University, New Taipei City 231, Taiwan

**Keywords:** COPD, triple therapy, single inhaler device, randomized controlled trials, mortality, dual therapy

## Abstract

There are more single inhaler device triple therapy available for COPD patients now. However, the effect of long-term triple therapy fixed dose combination (FDC) on mortality remains unclear. This study aimed to evaluate the impact of one-year single inhaler device triple therapy, including long-acting β2-agonists (LABAs), long-acting muscarinic receptor antagonists (LAMAs), and inhaled corticosteroids (ICSs), with dual therapies, comprised of either LABA/LAMA or ICS/LABA, on the mortality of patients with COPD. We searched the PubMed, Cochrane library, Web of Science, Embase databases, and clinical trial registry of clinicaltrials.gov and WHO ICTRP. Randomized controlled trials (RCTs) compared single inhaler device triple and dual therapies for 52 weeks were selected for the meta-analysis. The primary endpoint was all-cause mortality. A total of 6 RCTs were selected for the meta-analysis, including 10,274 patients who received single inhaler device triple therapy (ICS/LABA/LAMA FDC) and 12,395 patients who received ICS/LABA or LABA/LAMA dual therapy. Risk of death was significantly lower in the ICS/LABA/LAMA FDC group compared to the LABA/LAMA group (RR = 0.69, 95% CI = 0.53–0.90, *p* = 0.007). There was no significant difference in mortality between the ICS/LABA/LAMA FDC and ICS/LABA therapy groups (RR = 0.94, 95% CI = 0.72–1.24, *p* = 0.66). In addition, patients receiving ICS/LABA/LAMA FDC therapy had less moderate or severe exacerbations compared with the dual therapy groups (RR = 0.76, 95% CI = 0.73–0.80, *p* < 0.001 for LABA/LAMA; RR = 0.84, 95% CI = 0.78–0.90, *p* < 0.001 for ICS/LABA). By contrast, the risk of pneumonia in the ICS/LABA/LAMA FDC group was higher than in the LABA/LAMA group (RR = 1.43, 95% CI = 1.21–1.68, *p* < 0.001). In conclusion, ICS/LABA/LAMA FDC therapy could help improve the clinical outcomes of patients with COPD. However, triple therapy could increase the risk of pneumonia in comparison with LABA/LAMA dual therapy.

## 1. Introduction

The prevalence of chronic obstructive pulmonary disease (COPD) remains high and can be associated with high morbidity and mortality rates, so this clinical entity remains a major public health issue. However, COPD is a treatable disease, which is characterized by progressive airflow limitation [1]. In the view of pathophysiology, the inflammatory response plays an important role and can involve the airways, lung parenchyma, and pulmonary vasculature [2]. The chronic inflammation is caused by the prolonged exposure to noxious particles or gases and cigarette smoke remains the most common risk factor [1,2,3]. The inflammatory response and obstruction of the airways further cause a decrease in the forced expiratory volume (FEV_1_) and tissue destruction leads to airflow limitation and impaired gas exchange [3]. For COPD patients with continued dyspnea or at a higher risk of exacerbation due to an increased inflammation and air trapping under dual bronchodilator therapy of inhaled corticosteroids (ICSs) and long-acting β2-agonists (LABAs), or LABAs plus long-acting muscarinic receptor antagonists (LAMAs), triple inhaled therapy comprised of ICS/LABA/LAMA is suggested. [4,5,6,7] Before the development of a single inhaler device containing ICS, LABA and LAMA, open triple therapy, such as ICS plus LABA/LAMA or ICS/LABA plus LAMA was the most common prescription for patients who required triple therapy. Therefore, these patients needed at least two inhalers for use more than once daily, and these inhalers were used in different ways. There have been issues reported that were potentially associated with the high risk of incorrect use and poor compliance [8,9,10,11].

Recently, several triple fixed-dose combinations (FDCs) have been developed for COPD patients to use where ICS/LABA/LAMA are delivered simultaneously by a single inhaler device. A single device showed improvements in adherence and cost savings, [12,13,14,15] and their long-term use has been shown to be tolerable. [16,17,18,19] Currently, there are three different formulations for single inhaler device triple therapy: fluticasone furoate (FF)/vilanterol (VI)/umeclidinium (UMEC) (Trelegy Ellipta; GSK, Uxbridge, UK), [20] extra-fine beclomethasone dipropionate (BDP)/formoterol fumarate (FOR)/glycopyrronium bromide (GB) (Trimbow; Chiesi, Parma, Italy), [21,22,23] and budesonide (BUD)/FOR/GB (Aerosphere; Luton, UK). [24] Multiple randomized controlled trials (RCTs) exhibited positive clinical outcomes in COPD patients. [20,21,22,23,24,25] 52 weeks of extra-fine BDP/FOR/GB regimen had a lower rate of moderate-to-severe COPD exacerbations, [21,22,23] as compared with LAMA, ICS/LABA, LABA/LAMA, and open triple therapy. Similar findings of less moderate or severe exacerbations were observed in 52 weeks single inhaler device triple therapy with FF/VI/UMEC and BUD/FOR/GB compared with dual therapy, for moderate-to-very-severe COPD patients. [20,24] In the IMPACT trial, [20,26] all-cause mortality was significantly lower following administration of FF/VI/UMEC treatment regimens compared with VI /UMEC treatment (hazard ratio [HR], 0.58: 95% confidence intervals [CI], 0.38 to 0.88). In 2020, the ETHOS trial showed mortality benefit in 320 μg BUD/FOR/GB regimen as compared with FOR/GB therapy (HR, 0.54; 95% CI, 0.34 to 0.87). [24] However, the mortality benefit of triple FDC over dual therapy was not demonstrated in previous meta-analyses when the ETHOS trial was not included. [27,28,29] Therefore, an updated meta-analysis to investigate the impact of single device triple therapy versus dual therapy on the mortality of COPD patients is needed. In addition, the current study only evaluated the effect of 52 weeks triple therapy, to avoid the confounding factors associated with short treatment durations. The present study conducted a systematic review and meta-analysis of the previous literature to determine the effect of 52 weeks single inhaler device triple therapy compared with dual therapy (LABA/LAMA or ICS/LABA) on all-cause mortality in patients with COPD. Additional relevant outcomes including COPD exacerbation, change in lung function, quality of life, and risk of adverse events were assessed in this meta-analysis.

## 2. Methods

### 2.1. Study Search and Selection

This systematic review and meta-analysis of the literatures was performed and reported following Preferred Reporting Item for Systemic Reviews and Meta-Analyses (PRISMA) guidelines. [30] The protocol was registered at PROSPERO prespecified (reference number: CRD42020216746).

We searched for articles in the PubMed, Cochrane library, Web of Science, and Embase databases from their inception to 6 July 2021. The clinical trial registry of clinicaltrials.gov and WHO International Clinical Trials Registry Platform (ICTRP) were also searched. The search terms included COPD, triple-combination, LAMA (including tiotropium, aclidinium, umeclidinium, glycopyrronium, and glycopirrolate), LABA (including salmeterol, formoterol, vilanterol, olodaterol, and indacaterol), and ICS (including beclomethasone, fluticasone, budesonide, and ciclesonide) were used. The detail of search strategy was described in Supplementary Table S1. Reference list of recent published relevant reviews and meta-analysis were also examined for further proper literature. There was no language restriction applied.

The inclusion criteria for selection were as follows: (1) studied patients with COPD; (2) prospective, double blinded RCT; (3) single inhaler device triple therapy comprised of ICS, LABA, and LAMA as the intervention; (4) dual therapies comprised of either LABA/LAMA or ICS/LABA as the comparison; (5) a study outcome of mortality; (6) follow-up period for at least 52 weeks. Two authors (C.-C. Lai and C.-H. Chen) screened and selected publications independently to avoid any bias. They discussed with the third author (C.-Y. Wang) to make conclusion if they had different viewpoints.

Data were extracted from each included study, including publication year, design of study, location and duration, demographic characteristics of the including subjects, study and comparative therapy, outcome, and adverse events. Cochrane Risk of Bias Tool was used to evaluate the risk of bias and the quality of enrolled RCTs [31].

### 2.2. Definitions and Outcomes

The primary outcome was all-cause mortality. To comprehensive assess the efficacy and safety of single inhaler device triple therapy, the annual rate of moderate/severe COPD exacerbations, changes in the trough FEV_1_ in lung function from baseline, the change in the St. George’s Respiratory Questionnaire (SGRQ) from baseline, the risk of pneumonia, respiratory tract infection, adverse events, and cardiovascular events, were measured as secondary outcomes.

### 2.3. Statistical Analysis

Risk ratio (RR) and 95% CIs were used as the effect size measure for the primary outcome and other categorical outcomes. A RR > 1 indicates that the risk for moderate/severe COPD exacerbations or mortality was higher in patients with triple therapy than those with dual therapy, while a RR < 1 indicates that the risk is lower in the triple therapy group. For continuous outcomes (i.e., changes in SGRQ and FEV_1_), mean differences (MD) was considered as the measure of effect size. Greater improvements were indicated by positive values of MD for FEV_1_ and by negative values of MD for SGRQ.

Pooled estimates of the RRs and MDs across studies were calculated by DerSimonian–Laird random-effects model. Separate analyses were also performed according to the subgroups of the comparison group (i.e., dual therapy for LAMA/LABA or ICS/LABA). A two-sided *p*-value < 0.05 was considered as statistically significant. Study heterogeneity was presented using χ^2^-based Cochran’s Q statistic and I^2^. Cochran’s Q was defined by summing the square of the amount that each study’s estimate deviated from the overall estimate. For the Q statistic, *p* < 0.10 was considered statistically significant for heterogeneity. The I^2^ statistic indicated the percentage of the observed between-study variability that was due to heterogeneity. To evaluate if individual studies had large influences on the magnitude of the association between triple therapy and study outcomes, leave-one-out sensitivity analyses were conducted for each outcome. In addition, meta-regression was performed [32] to examine whether the association may differ by proportion of patients with higher eosinophil counts. Publication bias was presented by funnel plots and was assessed by egger’s test for all-cause mortality. Review Manager (RevMan) version 5.3 and Comprehensive Meta-Analysis, version 2.0 (Biostat, Englewood, NJ, USA) were used for statistical analyses.

## 3. Results

### 3.1. Study Basic Characteristics

**Figure 1:**
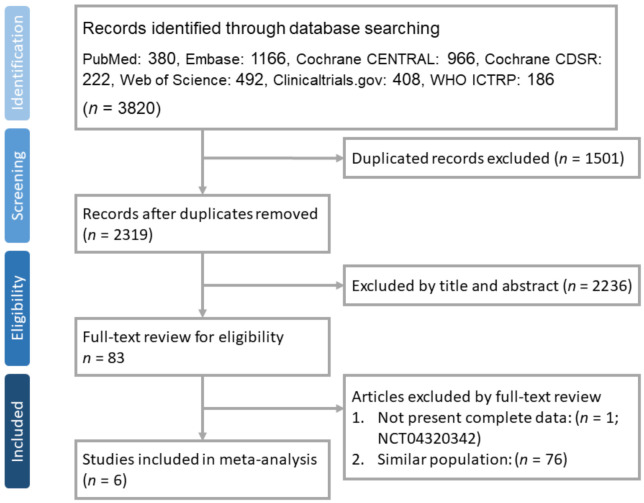
PRISMA flow diagram for selection of RCTs.

Our search yielded 3820 results in total, 380 from PubMed, 1166 from Embase, 492 from Web of Science core collection, 966 from Cochrane Central Trials databases, 222 from Cochrane Database of Systematic Reviews (CDSR) library, 408 from clinicaltrials.gov, and 186 from WHO ICTRP. After excluding 1501 duplicates, we carefully read the titles and abstracts of the remaining 2319 studies and 83 articles were selected for a full-text review. Finally, 6 studies [20,21,22,24,33,34] fulfilled the inclusion criteria and were used for further analysis (Figure 1). These 6 RCTs [20,21,22,24,33,34] included 10,274 patients who received triple therapy and 12,395 patients who received LABA/LAMA or ICS/LABA dual therapy. Two RCTs [20,32] did not exclude asthma patients. One study [24] recruited patients who received different doses of budesonide as part of their triple therapy (320 μg or 160 μg), and thus they were divided into two cohorts for subsequent meta-analyses. All patients received fixed triple therapy or dual therapy and were followed up for 52 weeks. The inclusion and exclusion criteria for each included study are described in Table 1.

### 3.2. Outcome Measures

**Figure 2:**
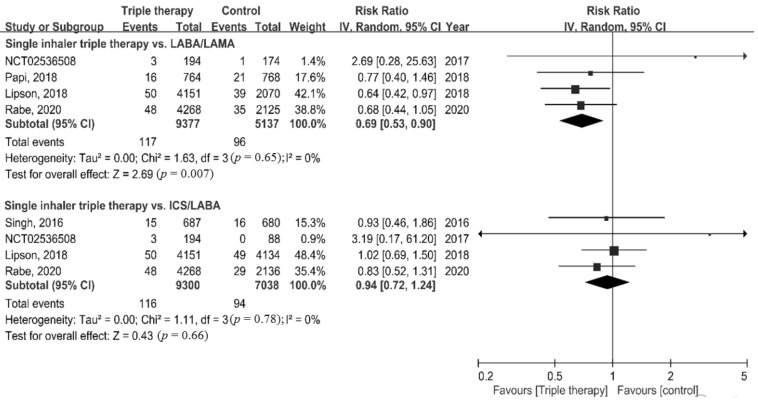
Forest plots for association of triple therapy with all-cause mortality.

Risk of death was significantly lower in the ICS/LABA/LAMA FDC group compared to the LABA/LAMA group (RR = 0.69, 95% CI = 0.53–0.90, *p* = 0.007). By contrast, no significant difference in mortality was found between the ICS/LABA/LAMA FDC group and the ICS/LABA group (RR = 0.94, 95% CI = 0.72–1.24, *p* = 0.66) (Figure 2).

**Figure 3:**
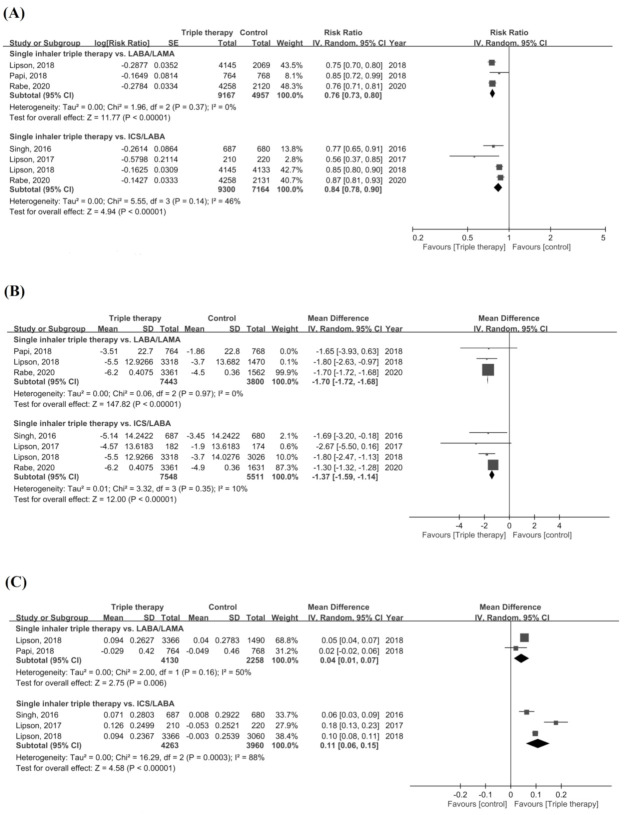
Forest plots for secondary outcomes. (**A**) Annual rate of moderate or severe COPD exacerbation, (**B**) change of SGRQ, and (**C**) change of FEV1.

For secondary outcomes, patients receiving ICS/LABA/LAMA FDC therapy had a significantly lower rate of moderate or severe exacerbations compared with LABA/LAMA or ICS/LABA dual therapy (RR = 0.76, 95% CI = 0.73–0.80, *p* < 0.001 for LABA/LAMA; RR = 0.84, 95% CI = 0.78–0.90, *p* < 0.001 for ICS/LABA) (Figure 3A). A significant improvement in SGRQ was observed in the single inhaler device triple therapy group compared with the dual therapy group (MD = −1.70, 95% CI = −1.72–−1.68, *p* < 0.001 for LABA/LAMA; MD = −1.37, 95% CI = −1.59–−1.14, *p* < 0.001 for ICS/LABA) (Figure 3B). ICS/LABA/LAMA FDC was associated with a significantly improved FEV_1_ compared with the two dual therapy groups (MD = 0.04, 95% CI = 0.01–0.07, *p* = 0.006 for LABA/LAMA; MD = 0.11, 95% CI = 0.06-0.15, *p* < 0.001 for ICS/LABA) (Figure 3C). However, high significant heterogeneity was observed in assessment of annual rate of moderate or severe exacerbations (*p* = 0.03, *I^2^* = 78.6%), the change in the SGRQ score (*p* = 0.004, I^2^ = 88.2%), and the change in FEV_1_ (*p* = 0.02, I^2^ = 80.9%).

**Figure 4:**
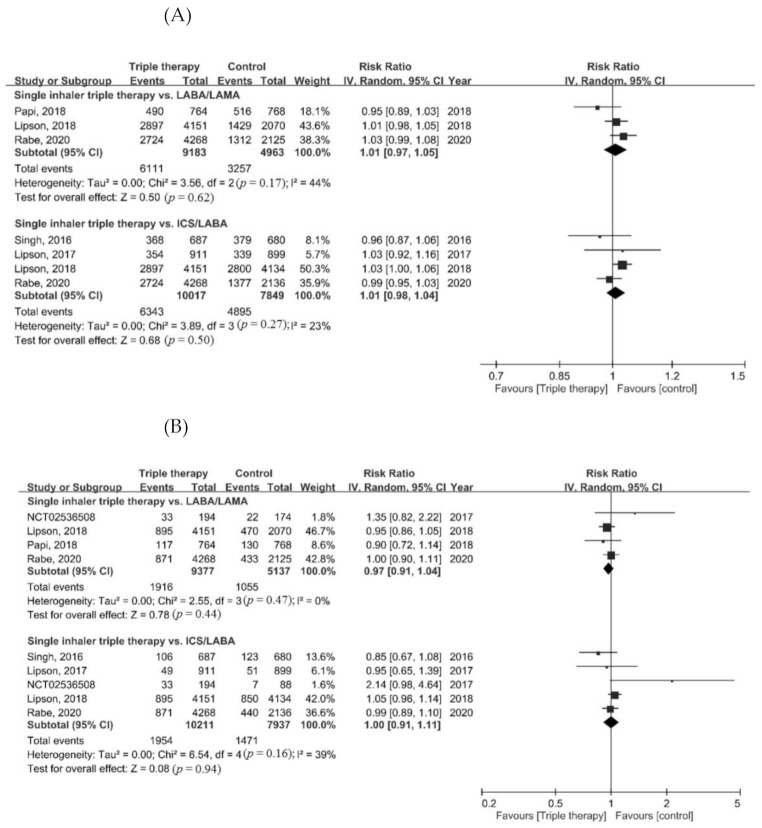

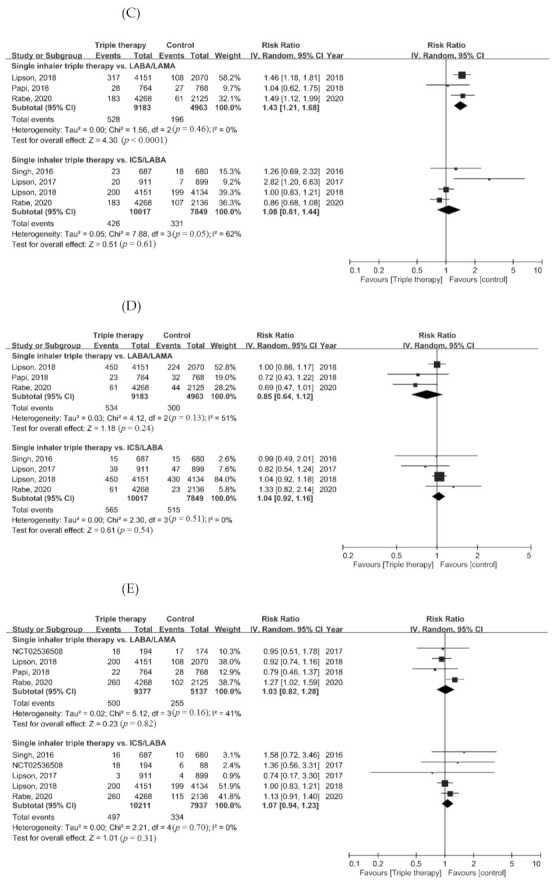
Forest plots for secondary outcomes. (**A**) Adverse event, (**B**) serious adverse event, (**C**) pneumonia, (**D**) cardiovascular event, and (**E**) respiratory tract infection.

Regarding the risk of adverse events, the risk of pneumonia in the ICS/LABA/LAMA FDC group was higher than in the LABA/LAMA group (RR = 1.43, 95% CI = 1.21–1.68, *p* < 0.001). There was no difference in the risk of adverse events, serious adverse events, cardiovascular events, and respiratory tract infections between the ICS/LABA/LAMA FDC group and the dual therapy groups (Figure 4A–E).

### 3.3. Sensitivity Analyses

Results of the leave-one-out sensitivity analysis showed that the magnitude of each study outcome associated to ICS/LABA/LAMA FDC therapy was not influenced by individual studies.

### 3.4. Meta-Regression

Four studies provided data pertaining to proportion of high baseline eosinophil, despite the cut-off value was different (the cut-off point was 200 cells/mm^3^ in three studies [20,21,22] and 150 cells/mm^3^ in one study [24]). The results of meta-regression analysis showed that the eosinophil counts at baseline may affect the impact of ICS/LABA/LAMA FDC therapy on the primary and secondary outcomes association of (Table 2). In terms of mortality, the reduction effect of ICS/LABA/LAMA FDC therapy, compared to LABA/LAMA, was more prominent in studies in which more patients with high eosinophil counts at baseline (slope −0.74, *p* = 0.006). Besides, studies with more COPD patients with higher eosinophil count have lower rate of exacerbation in ICS/LABA/LAMA FDC therapy, either compared to ICS/LABA (slope −0.31, *p* < 0.001) or LABA/LAMA (slope −0.50, *p* < 0.001). There was also more reduction of SGRQ score in studies with more patients of higher eosinophil count with ICS/LABA/LAMA FDC therapy, than with ICS/LABA (slope −2.67, *p* < 0.001) or LABA/LAMA (slope −3.09, *p* < 0.001). However, more patients with higher eosinophil count using ICS/LABA/LAMA FDC therapy would have higher risk of pneumonia as compared to using LABA/LAMA (slope 0.71, *p* < 0.001).

### 3.5. Quality Assessment

**Figure 5:**
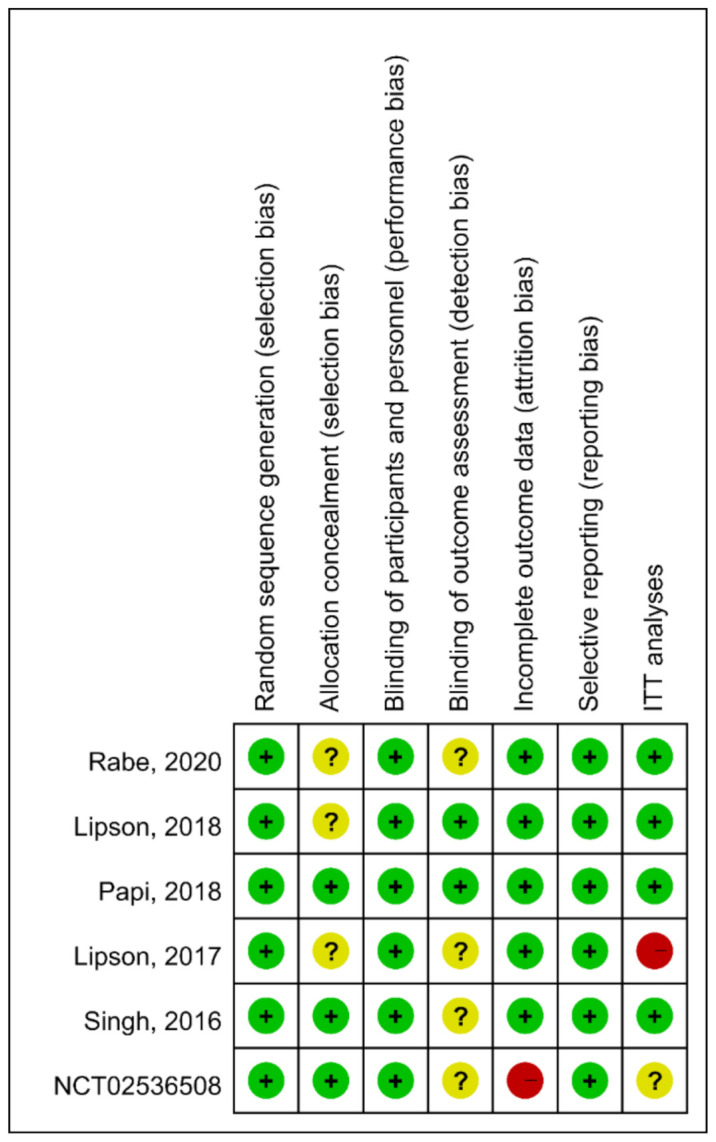
The summary of risk of bias. Green (+): low risk; Yellow (?): unknown risk; Red (-), high risk.

Risk of bias for the included studies is depicted in Figure 5. Instead of intention-to-treat population, one study [33] was analyzed with an extension population followed by 52 weeks. Some studies did not describe procedures regarding allocation concealment and assessment of outcome blinding in detail. One study [34] carried a high risk of attrition bias due to higher proportion (26.1%) of patient withdraw, discontinuation due to adverse event, and/or loss to follow up.

### 3.6. Publication Bias

**Figure 6:**
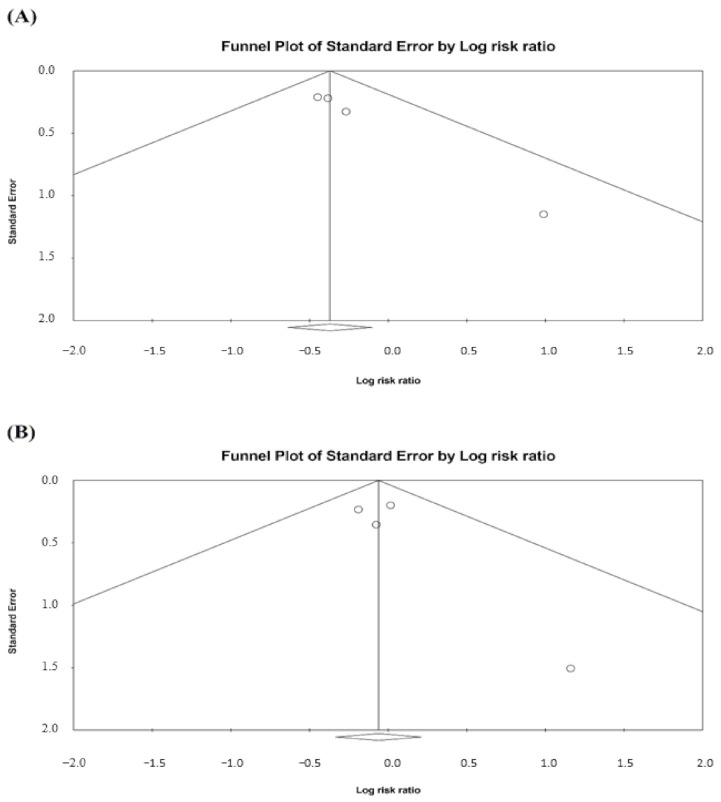
Funnel plots for mortality. (**A**) Triple therapy versus LAMA/LABA and (**B**) triple therapy versus ICS/LABA. A circle symbol presents an individual study.

The funnel plots for mortality were presented in Figure 6. Publication bias was observed for the association with mortality between triple therapy and LAMA/LABA (Egger’s test: t = 9.95, *p* = 0.010), but not for triple therapy versus ICS/LABA (Egger’s test: t = 1.13, *p* = 0.375).

## 4. Discussion

This meta-analysis investigated the impact of 52 weeks single inhaler device triple therapy versus dual therapy on COPD patient clinical outcomes, and it identified significant differences between the two therapies. First, compared with LABA/LAMA, triple therapy was associated with a lower mortality but no significant difference in the risk of death was observed between triple therapy and ICS/LABA. Second, triple therapy was associated with a better quality of life and lung function that both LABA/LAMA and ICS/LABA. Finally, triple therapy carried a higher risk of pneumonia than LABA/LAMA. Most importantly, this meta-analysis demonstrated that single inhaler device triple therapy was associated with a significantly lower mortality rate compared with dual LABA/LAMA therapy. This contrasted with previous meta-analyses, [27,28,29] which showed that the risk of death among ICS/LABA/LAMA FDC patients was numerically lower compared with LABA/LAMA therapy patients, but that the difference was not statistically significant. The differences between this study and previous studies [27,28,29] can be explained as follows: (1) this meta-analysis included the ETHOS trial [24] which is a recent trial and was not investigated in previous meta-analyses; the ETHOS trial included 8509 patients and found that high-dose BUD/FOR/GB had a mortality benefit compared with LABA/LAMA; (2) in contrast to previous meta-analyses, [27,28,29] in which the study duration varied from 24 weeks to 52 weeks, we only enrolled studies which investigated 52 weeks of triple therapy, to assess the long-term effects; (3) the largest study, the IMPACT trial [20], which included 10,355 patients, also reported lower all-cause mortality rates for triple therapy compared with dual therapy, so the findings of the ETHOS and IMPACT trials [20,24], which comprised most of the patients in this meta-analysis may have determined the overall results of this study. Overall, our findings which were based on a meta-analysis of a large number of patients, indicated that single inhaler device triple therapy for one year could provide additional mortality benefits compared with LABA/LAMA dual therapy.

By contrast, although the risk of death among ICS/LABA/LAMA FDC users was numerically lower than those using ICS/LABA dual therapy, the difference was not statistically significant. This finding is consistent with previous studies, [27,28,29] in which the study duration varied, ranging from 12 to 52 weeks. Therefore, our findings confirmed that ICS/LABA/LAMA FDC therapy cannot significantly improve the mortality of COPD patients using ICS/LABA treatment, even after use for one year.

In addition, another important outcome: the risk of moderate or severe COPD exacerbations, was evaluated in the present study. We found that ICS/LABA/LAMA FDC therapy was associated with a lower rate of exacerbations compared with patients using LABA/LAMA or ICS/LABA dual therapy. Moreover, ICS/LABA/LAMA FDC users also had a significantly improved quality of life and lung function in terms of changes in their SGRQ score and FEV_1_ compared with LABA/LAMA or ICS/LABA dual therapy. However, these findings should be interpreted cautiously because these improvements did not meet the definition about the SGRQ minimal important difference (MID) of SGRQ of 4 points and FEV_1_ of 100 mL. Overall, all these findings are consistent with the findings of previous meta-analyses, [27,28,29,35] and suggest that the use of ICS/LABA/LAMA FDC therapy could help reduce the risk of moderate or severe exacerbations and improve lung function and quality of life among COPD patients.

Regarding eosinophil level, some previous studies reported that the eosinophil count or percentage of eosinophils can predict COPD outcomes [36]. Regarding the risk of COPD exacerbation, the protective effect of triple combination therapy versus LABA/LAMA dual therapy was greater in patients with blood eosinophil counts ≥ 300 cells per μL [33]. However, in the previous meta-analysis by Calzetta et al., [32] showed that the protective effect of ICS/LABA/LAMA combination therapy versus ICS/LABA or LABA/LAMA dual therapy, against the risk of mortality, moderate or severe AE COPD or pneumonia, was not associated with eosinophil levels. In our meta-regression analysis, we found that the patients with higher eosinophil count have decreased mortality, COPD AE and SGRQ score but more risk of pneumonia with using triple FDC therapy than LABA/LAMA. They also have decreased COPD AE and SGRQ score with using triple FDC therapy than LABA/ICS. COPD with higher blood eosinophil count would increase the risk of pneumonia hospitalizations has been notice by Vedel-Krogh et al. [37], but the risk of pneumonia seems not diminished the benefit in mortality of triple FDC therapy. Although additional ICS to LABA/LAMA in higher eosinophil patients was already suggested [38], addition LAMA to ICS/LABA may be also considered in higher eosinophil patients according to our findings. Further studies are warranted to clarify this issue.

Finally, the risk of adverse events associated with ICS/LABA/LAMA FDC therapy remains another serious concern. Although there was no significant difference regarding the risk of adverse events, serious adverse events, cardiovascular events and respiratory tract infections between triple therapy and dual therapy (LABA/LAMA or ICS/LABA), we found that ICS/LABA/LAMA FDC therapy had significantly higher risk of pneumonia compared with LABA/LAMA therapy (RR = 1.42, 95% CI = 1.21–1.66, *p* < 0.001) in the pooled analysis of 3 RCTs, [20,21,24] in which BUD, FF, and BDP were used as the ICS, one in each study. This was consistent with previous studies [27,28,29] which reported that there was a higher risk of pneumonia with triple therapy compared with LABA/LAMA therapy, and it reminded clinicians of the possible development of pneumonia among COPD patients who are using triple therapy. This finding could be explained by that ICSs exhibit both anti-inflammatory and immunosuppressive effects, which might further cause the impairment of pulmonary and/or systemic host defense. In addition, an inflammatory response is mounted against invading pathogens, so ICS’s anti-inflammation effect may increase the risk of respiratory infections, especially in subjects with impaired immune system in the airways and lungs, such as COPD [39,40,41].

This meta-analysis had three major strengths. First, it provided updated information after the addition of the recent ETHOS trial, which was just published in July 2020. Second, to avoid the confounding effects of study duration, only studies which investigated the use of single inhaler device triple therapy for a minimum of one year were included in this meta-analysis. Third, our data analyzed the effect of eosinophil for choosing triple therapy or other therapies, which was not discussed in other meta-analysis. [42,43]

### Limitations

There are some limitations in this meta-analysis. First, although most of the studies only enrolled patients with an FEV_1_ <50%, some of the studies did not. Second, patients included in the analysis used different ICS/LABA/LAMA combinations, and the triple combination, LABA/LAMA and ICS/LABA were administered from different devices and in different dosing regimens. Furthermore, the results of meta-regression should be interpreted in caution. Since proportion of high eosinophil counts at baseline, which is a study-level variable for blood eosinophil, was used in the analysis, these study-level results may not appropriate to apply to individual patients with higher eosinophils. Nonetheless, our results were consistent to other patient-level study [44], the bias regarding “ecological fallacy” might be compromised. Besides, the data also imply the importance of checking eosinophils level for COPD patients. Finally, the number of enrolled RCTs was limited in this meta-analysis, particularly because each had unique treatment arm comparisons. All these factors may affect the heterogeneity of the meta-analysis.

## 5. Conclusions

This meta-analysis indicated that COPD patients with ICS/LABA/LAMA FDC single inhaler device triple therapy could reduce 31% mortality rate compared to those with LABA/LAMA dual therapy. In addition, ICS/LABA/LAMA FDC therapy can also result in 24% and 16% lower rate of moderate or severe COPD exacerbations than LABA/LAMA and ICS/LABA, respectively. Moreover, ICS/LABA/LAMA FDC therapy is also associated with better lung function and quality of life compared with LABA/LAMA or ICS/LABA dual therapy. However, more pneumonia was found in triple therapy as compared with LABA/LAMA dual therapy. Therefore, more attention should be paid to the risk pneumonia while using ICS/LABA/LAMA FDC therapy in order to obtain a better outcome in COPD moderate or severe exacerbations and mortality.

## Figures and Tables

**Table 1:** Characteristics of enrolled studies.

Study	Study Site	No of Participants	Study Period	Inclusion Criteria					Inhalation Therapy		Primary Outcome
				FEV1	Exacerbation history in previous year	Symptom scores	Excluded asthma	Others	Fixed triple	Comparator	
Lipson et al., 2018 (IMPACT) [20]	37 countries	10,355	2014–2017	FEV_1_ of 50-80%	≥1 moderate/severe exacerbation if FEV_1_ < 50% or ≥2 moderate exacerbations or one severe exacerbation if FEV1 of 50-80%	CAT score ≥ 10	No	≥40 years; MCID: 2 point; use LAMA, a LABA, or an ICS alone or in combination	FF/UME/VIL	FF/VIL or UME/VIL	Annual rate of moderate or severe COPD exacerbations
Papi et al., 2018 (TRIBUTE) [21]	187 sites in 17 countries	1532	2015–2017	FEV1 < 50%	≥1 moderate or severe exacerbation	CAT score ≥ 10	Yes	≥40 years; current or ex-smoker; used ICS/LABA, ICS/LAMA or LABA/LAMA for ≥2 months	BDP/FOR/GB	IND/GB	Moderate to severe COPD exacerbation rate for 52 weeks
Singh et al., 2016 (TRIOLOGY) [22]	159 sites in 14 countries	1368	2014–2016	FEV_1_ < 50%	≥1 moderate/severe exacerbation	CAT score ≥ 10	Yes	≥40 years; current or ex-smoker; used ICS/LABA, ICS/LAMA or LABA/LAMA for ≥2 months	BDP/FOR/GB	BDP/FOR	Moderate to severe COPD exacerbation rate for 52 weeks
Rabe et al., 2020 (ETHOS) [24]	740 sites in 26 countries	8509	2015–2019	FEV1 of 25-65%;	≥1 moderate/severe exacerbation if FEV1 < 50% or ≥2 moderate exacerbations or one severe exacerbation if FEV1 ≥ 50%	CAT score ≥ 10	Yes	40 to 80 years; MICD: 2 point; receiving at least two inhaled maintenance therapies at the time of screening; a smoking history of at least 10 pack-years	BUD/FOR/GB	GB/FOR or BUD/FOR	Annual rate of moderate or severe COPD exacerbations
Lipson et al., 2017 (FULFIL)—extension population [33]	160 sites in 15 countries	430	2015–2016	FEV_1_ < 50% or 50%-80%	≥2 moderate exacerbations or ≥1 severe exacerbation if FEV1 ≥ 50%	CAT score ≥ 10	Yes	≥40 years; receiving daily maintenance therapy for COPD for at least 3 months	FF/UME/VIL	BUD/FOR	Lung functionand health-related quality of life
NCT02536508 [34]	64 cites in US	627	2015–2017	NA	NA	NA	No	40 to 80 years, moderate to very severe COPD	BUD/FOR/GB	GB/FOR or BUD/FOR	Percent change from baseline in BMD of the lumbar spine

BDP, beclometasone dipropionate; BMD, bone mineral density; FOR, formoterol fumarate, GB, glycopyrronium; IND, indacaterol; TIO, tiotropium; UME, umeclidinium; VIL, vilanterol; BUD, budesonide; FF, fluticasone furoate; MCID, minimum clinically important difference; COPD Assessment Test, CAT; inhaled corticosteroid, ICS; long-acting b2-agonist, LABA; long-acting muscarinic antagonist, LAMA.

**Table 2:** Meta-regression analysis with proportion of patients with higher eosinophil counts for identification of effect modification of COPD-related covariates on the association between triple therapy and study outcomes in COPD patients.

	ICS/LABA		LAMA/LABA	
Outcome	Slope	*p*	Slope	*p*
Mortality	−0.16	0.545	−0.74	0.006
Annual rate of moderate/severe exacerbation	−0.31	<0.001	−0.50	<0.001
Change of SGRQ	−2.67	<0.001	−3.09	<0.001
Change of FEV1	NA		NA	
Adverse event	0.00	0.997	0.03	0.272
Serious adverse event	−0.01	0.911	−0.05	0.421
Pneumonia	−0.10	0.479	0.71	<0.001
Cardiovascular event	0.14	0.328	−0.36	0.157
Respiratory tract infection	0.16	0.247	0.11	0.673

## Supplementary Materials

The following are available online at https://www.mdpi.com/article/10.3390/life12020173/s1, Table S1: Search strategy.
