# Supplementary material for: The Impact of 52-Week Single Inhaler Device Triple Therapy versus Dual Therapy on the Mortality of COPD Patients: A Systematic Review and Meta-Analysis of Randomized Controlled Trials
Source: Life (Basel). 2022 Jan 25;12(2):173. doi: 10.3390/life12020173 (PMC8877713; doi:10.3390/life12020173)
Supplement: Supplementary file 1 [file life-12-00173-s001.zip › life-1505690-supplementary.pdf]

Supplementary Table S1. Search strategy

Search strategy in **Pubmed** (Search date: 20210706)

PICO	Search	Query	Items found	Time
	39	#33 AND #38	380	03:56:31
	38	#34 OR #35 OR #36 OR #37	1,480,696	03:56:25
	37	random*	1,475,518	03:56:19
	36	RCT	27,207	03:56:14
	35	Randomized Controlled Trial*	793,860	03:56:10
	34	Randomized Controlled Trial [MeSH term]	712,927	03:56:04
**PI**	33	#4 AND #32	1,119	03:55:58
**I**	32	#7 OR #31	91,056	03:55:54
	31	#15 AND #23 AND #30	1,031	03:55:48
	30	#24 OR #25 OR #26 OR #27 OR #28 OR #29	36,219	03:55:42
	29	Ciclesonide	424	03:55:37
	28	Budesonide	6,637	03:55:31
	27	Fluticasone	4,817	03:55:26
	26	Beclomethasone	3,967	03:55:22
	25	inhaled corticosteroid	17,602	03:55:17
	24	ICS	13,407	03:55:12
	23	#16 OR #17 OR #18 OR #19 OR #20 OR #21 OR #22	8,529	03:55:08
	22	Indacaterol	529	03:55:02
	21	Olodaterol	236	03:54:56
	20	Vilanterol	437	03:54:49
	19	Formoterol	2,695	03:54:43
	18	Salmeterol	3,070	03:54:37
	17	long-acting beta agonist	4,080	03:54:30
	16	LABA	2,146	03:54:25
	15	#8 OR #9 OR #10 OR #11 OR #12 OR #13 OR #14	6,877	03:54:17
	14	Glycopirrolate	1	03:54:11
	13	Glycopyrronium	1,736	03:54:04
	12	Umeclidinium	284	03:53:57

	11	Aclidinium	236	03:53:50
	10	Tiotropium	1,901	03:53:42
	9	long-acting muscarinic receptor antagonist	1,062	03:53:32
	8	LAMA	3,251	03:53:25
	7	#5 OR #6	90,315	03:53:17
	6	Triple-combination	1,736	03:53:09
	5	Triple	90,315	03:53:02
**P**	4	#1 OR #2 OR #3	94,358	03:52:54
	3	Pulmonary Disease, Chronic Obstructive [MeSH term]	86,939	03:52:46
	2	Chronic Obstructive Pulmonary Disease	86,939	03:52:39
	1	COPD	94,358	03:50:37

Search strategy in **Embase** (Search date: 20210706)

	No.	Query	Results
**PI**	#37	#4 AND #32 AND #36	1166
**I**	#36	#33 OR #34 OR #35	1924229
	#35	random*	1912561
	#34	rct	47884
	#33	'randomized controlled trial'/exp [Emtree term]	667168
	#32	#7 OR #31	136606
	#31	#15 AND #23 AND #30	4025
	#30	#24 OR #25 OR #26 OR #27 OR #28 OR #29	79908
	#29	ciclesonide	1750
	#28	budesonide	24435
	#27	fluticasone	19461
	#26	beclomethasone	9777
	#25	inhaled AND corticosteroid	19535
	#24	ics	31498
	#23	#16 OR #17 OR #18 OR #19 OR #20 OR #21 OR #22	22300
	#22	indacaterol	1910
	#21	olodaterol	790
	#20	vilanterol	1499
	#19	formoterol	9711
	#18	salmeterol	12111
	#17	'long-acting beta agonist'	866
	#16	laba	5011
	#15	#8 OR #9 OR #10 OR #11 OR #12 OR #13 OR #14	20359
	#14	glycopirrolate	5
	#13	glycopyrronium	7814
	#12	umeclidinium	1023
	#11	aclidinium	870
	#10	tiotropium	6769
	#9	'long-acting muscarinic receptor antagonist'	85
	#8	lama	6892
	#7	#5 OR #6	133306
	#6	triple	133306
	#5	'triple combination'	3076
**P**	#4	#1 OR #2 OR #3	173676
	#3	'chronic obstructive pulmonary disease'	78721

	#2	copd	98972
	#1	'chronic obstructive lung disease'/exp [Emtree term]	147976

Search strategy in Cochrane Library (Search date: 20210706)

PICO	Search	Query
**P**	#1	COPD
	#2	Chronic Obstructive Pulmonary Disease
	#3	Pulmonary Disease, Chronic Obstructive [Mesh term]
	#4	#1 OR #2 OR #3
**I**	#5	Triple-combination
	#6	Triple
	#7	#5 OR #6
	#8	LAMA
	#9	long-acting muscarinic receptor antagonist
	#10	Tiotropium
	#11	Aclidinium
	#12	Umeclidinium
	#13	Glycopyrronium
	#14	Glycopirrolate
	#15	#8 OR #9 OR #10 OR #11 OR #12 OR #13 OR #14
	#16	LABA
	#17	long-acting beta agonist
	#18	Salmeterol
	#19	Formoterol
	#20	Vilanterol
	#21	Olodaterol
	#22	Indacaterol
	#23	#16 OR #17 OR #18 OR #19 OR #20 OR #21 OR #22
	#24	ICS
	#25	inhaled corticosteroid
	#26	Beclomethasone
	#27	Fluticasone
	#28	Budesonide
	#29	Ciclesonide
	#30	#24 OR #25 OR #26 OR #27 OR #28 OR #29
	#31	#15 AND #23 AND #30
	#32	#7 OR #31
**PI**	#33	#4 AND #32

Cochrane Database of Systematic Reviews (CDSR): 222

Cochrane Central Register of Controlled Trials (CENTRAL): 966

Search strategy in **Web of Science Core Collection** (Search date: 2021/07/06)

PICO	#	Query	Results
	#36	#31 AND #35	492
	#35	#32 OR #33 OR #34	1,869,344
	#34	ALL=(random*)	1,862,604
	#33	ALL=(RCT)	28,797
	#32	ALL=('Randomized Controlled Trial*')	446,587
**PI**	#31	#3 AND #30	1,452
	#30	#7 OR (#14 AND #22 AND #29)	11,169
	#29	#23 OR #24 OR #25 OR #26 OR #27 OR #28	56,153
	#28	ALL=(Ciclesonide)	534
	#27	ALL=(Budesonide)	10,004
	#26	ALL=(Fluticasone)	8,667
	#25	ALL=(Beclomethasone)	4,511
	#24	ALL=('inhaled corticosteroid')	15,000
	#23	ALL=(ICS)	31,218
	#22	#15 OR #16 OR #17 OR #18 OR #19 OR #20 OR #21	11,783
	#21	ALL=(Indacaterol)	886
	#20	ALL=(Olodaterol)	394
	#19	ALL=(Vilanterol)	670
	#18	ALL=(Formoterol)	4,004
	#17	ALL=(Salmeterol)	5,344
	#16	ALL=('long-acting beta agonist')	4,255
	#15	ALL=(LABA)	2,426
	#14	#7 OR #8 OR #9 OR #10 OR #11 OR #12 OR #13	14,087
	#13	ALL=(Glycopirrolate)	2
	#12	ALL=(Glycopyrronium)	791
	#11	ALL=(Umeclidinium)	426
	#10	ALL=(Aclidinium)	458
	#9	ALL=(Tiotropium)	3,294
	#8	ALL=('long-acting muscarinic receptor antagonist')	264
	#7	ALL=(LAMA)	10,257
	#6	#4 OR #5	178,968
	#5	ALL=('Triple-combination')	2,086
**I**	#4	ALL=(Triple)	178,968

	#3	#2 OR #1	93,433
	#2	ALL=(COPD)	70,090
**P**	#1	ALL=('Chronic Obstructive Pulmonary Disease')	61,490

Search strategy in **clinicaltrials.gov** (Search date: 2021/07/06)
Keyword:
Condition or disease: COPD
Other terms: Triple OR ((LAMA OR Tiotropium OR Aclidinium OR Umeclidinium OR Glycopyrronium) AND (LABA OR Salmeterol OR Formoterol OR Vilanterol OR Olodaterol OR Indacaterol) AND (ICS OR Beclomethasone OR Fluticasone OR Budesonide OR Ciclesonide))

Result: 408 trials

Search strategy in **WHO International Clinical Trials Registry Platform** (Search date: 2021/07/06)
Keyword: ('Chronic Obstructive Pulmonary Disease' OR COPD) AND (Triple OR ((LAMA OR Tiotropium OR Aclidinium OR Umeclidinium OR Glycopyrronium) AND (LABA OR Salmeterol OR Formoterol OR Vilanterol OR Olodaterol OR Indacaterol) AND (ICS OR Beclomethasone OR Fluticasone OR Budesonide OR Ciclesonide)))

Result: 186 trials
